# A one-year follow-up study of the Symbol Digit Modalities Test (SDMT) and the Paced Auditory Serial Addition Test (PASAT) in relapsing-remitting multiple sclerosis: an appraisal of comparative longitudinal sensitivity

**DOI:** 10.1186/s12883-015-0296-2

**Published:** 2015-03-22

**Authors:** Mariana López-Góngora, Luis Querol, Antonio Escartín

**Affiliations:** Multiple Sclerosis Unit, Department of Neurology, Hospital de la Santa Creu i Sant Pau, Universitat Autònoma de Barcelona, Sant Antoni M. Claret 167, Barcelona, 08025 Spain; Departament de Medicina, Universitat Autònoma de Barcelona, Passeig de la Vall d’Hebron, 119-129, Barcelona, 08035 Spain; Multiple Sclerosis Research Group, Biomedical Research Institute (IIB-Sant Pau), Sant Antoni M. Claret 167, Barcelona, 08025 Spain

**Keywords:** Neuropsychological assessment, Multiple sclerosis, Attention, Speed of information processing, Executive function, Cognition

## Abstract

**Background:**

Neuropsychological batteries are infrequently used to assess cognitive impairment in multiple sclerosis because they are time-consuming and require trained personnel. The Symbol Digit Modalities Test (SDMT) is suggested to be a useful screening tool to measure cognitive impairment in multiple sclerosis patients and is more valid and reliable over time than the Paced Auditory Serial Addition Test (PASAT). The purpose of this study was to evaluate which of these tests was more sensitive to cognitive impairment at one-year follow-up.

**Methods:**

A total of 237 patients with relapsing-remitting multiple sclerosis and 57 healthy controls underwent a complete neuropsychological assessment. One year later, we assessed 196 patients using the Brief Repeatable Battery of Neuropsychological Tests. We also administered other executive function and prospective memory tests, together with fatigue and depression questionnaires.

**Results:**

A total of 33.8% of patients were classified as cognitively impaired. The SDMT and the PASAT 3 seconds test (PASAT3) had a sensitivity of 0.809 and 0.783, respectively, thereby classifying patients as cognitively impaired. Analysis of 196 patients one year later showed 31.6% had cognitive impairment compared with 27.6% at the first assessment. The sensitivity to detect cognitive impairment after one year was 0.824 for SDMT and 0.796 for PASAT3. When the predictors were removed from the comparative standard battery, SDMT still showed a slightly higher sensitivity. Both SDMT and PASAT3 correlated significantly with all tests, but SDMT showed higher correlation values. Furthermore, SDMT was completed by all subjects while PASAT3 was completed by 86.9% of patients and 94.7% of controls.

**Conclusions:**

SDMT is simpler to administer than PASAT3 and may be slightly more sensitive to MS cognitive impairment. It could thus be a suitable test to assess cognitive impairment routinely in people with relapsing-remitting multiple sclerosis.

## Background

Cognitive impairment (CI) in multiple sclerosis (MS) is common and disabling [1], affecting recent memory, sustained attention, verbal fluency, conceptual reasoning, and visuospatial perception [2]. It is present at all stages of the disease and limits work and social activities [3]. Because cognitive impairment plays a relevant role in MS, neuropsychological assessment in routine clinical practice is needed [1].

Neuropsychological batteries currently available to assess CI in MS patients are time-consuming and therefore are not administered to all patients. The Rao Brief Repeatable Neuropsychological Battery (BRNB) and the Minimal Assessment on Cognitive Function in MS (MACFIMS) are most commonly used and take 25–30 and 90 minutes, respectively, to administer [4,5]. Other batteries such as the Neuropsychological Screening Battery for Multiple Sclerosis (NPSBMS), the Screening Examination for Cognitive Impairment (SEFCI), and the Repeatable Battery for the Assessment of Neuropsychological Status (RBANS) take less time to administer, but still require at least 20 minutes [6]. However, a recently developed battery, the Brief International Assessment of Cognition for MS (BICAMS), takes 15 minutes to complete [7]. While the BICAMS was intended to require a minimum of training, the value of a single test for monitoring cognition in MS remains a valuable goal.

The Symbol Digit Modalities Test (SDMT) has been proposed for the assessment of CI in MS. SDMT measures patient attention, concentration and speed of information processing [8]. In a study of recently diagnosed MS patients, Deloire et al. [9] observed that it correctly classified 75.4% of patients. Parmenter et al. [10] found that a score of 55 or lower accurately classified CI in MS patients. Benedict et al. [11] found that the SDMT was valid for discriminating patients from controls even when it was administered each month over six months. In addition, it is simple to administer and takes only five minutes to complete [12].

Another screening test proposed for use in MS is the Paced Auditory Serial Addition Test (PASAT), an auditory test that also measures working memory. It measures the same cognitive functions as SDMT. PASAT is part of the Multiple Sclerosis Functional Composite (MSFC), developed to measure impairment and disability in MS [13], and it is widely used in clinical research. Despite its extensive use, Brooks et al. showed that results are inadequate even in young and intellectually active subjects [14].

In a recent study, Sonder et al. [15] reported that SDMT was more valid and more reliable over time than the PASAT3 seconds test (PASAT3) as a tool to assess cognition in MS patients. However, to our knowledge, no longitudinal, prospective studies have attempted to predict deficits in cognition.

The aim of our study was to evaluate and compare SDMT and PASAT3 to determine which was more sensitive for the detection of CI when compared with a complete neuropsychological evaluation in a group of patients with relapsing-remitting multiple sclerosis (RRMS) after one year of follow-up.

## Methods

### Participants

Consecutive patients fulfilling McDonald’s criteria for MS [16] and attending regular appointments at our MS unit were recruited from December 2008 to January 2013. Only patients with RRMS were included. Exclusion criteria were a progressive form of the disease, alcohol, or drug dependence, presence of a neurological disease other than MS, history of a medical or psychiatric disorder that could affect cognitive function, relapse, and corticosteroid treatment in the past month.

Two hundred and thirty-seven patients who met the inclusion criteria and 57 healthy controls recruited from friends and in-law relatives matched by age, gender, and education level agreed to participate in the study. Neuropsychological assessment with parallel forms of the tests was performed in one hundred and ninety-six patients one year later. All participants gave their written informed consent before entering the study. The study was approved by the ethics committee at the Hospital de la Santa Creu i Sant Pau and performed according to the Declaration of Helsinki.

### Clinical and neuropsychological examination

Neurological examination of all participants was performed by the same experienced MS neurologist. Relapses were evaluated and disability was assessed to determine the expanded disability status scale (EDSS) score [17].

Neuropsychological examination was always performed by the same clinical neuropsychologist. This examination included the Spanish version of the Brief Repeatable Battery of Neuropsychological Tests (BRB-N) [18], which consists of the Selective Reminding Test (SRT) for verbal memory acquisition and delayed recall, the Spatial Recall Test (10/36) for visual memory acquisition and delayed recall, the Symbol Digit Modalities Test (SDMT) and the Paced Auditory Serial Addition Test (PASAT) for sustained and complex attention, information processing speed and working memory, and the Word List Generation (WLG) for verbal fluency.

We used other tests to evaluate executive function: the Stroop Test [19], the Wisconsin Card Sorting Test (WCST) [20], and the matrix subtest from the Wechsler Adult Intelligence Scale (WAIS) [21]. We also assessed visuospatial functions using the Line Orientation Test (LO) [22]. For prospective memory, we used the Spanish translation of the Rivermead Behavioral Memory Test (RBMT) [23]. To assess depression and fatigue, we used the Beck Depression Inventory (BDI) [24] and the Fatigue Severity Scale (FSS) [25].

In accordance with previously published classifications [15,26], cognitive impairment was defined as a performance of ≤1.5 standard deviations below control subjects on two or more tests of the BRB-N. We based the scores for control subjects on a previously published study using the Spanish version of the BRB-N [27].

### Statistical analysis

We analyzed differences between the control group and patients regarding demographic variables and cognitive assessment results using a T test for independent samples. The same test was also used to compare demographic characteristics of patients who completed the PASAT3 with those who did not.

A Pearson correlation coefficient was used to study the correlation between SDMT and PASAT3 and also between these two tests and other cognitive variables. To avoid the influence of outliers, non-parametric correlations (Spearman correlation coefficient) were used. We applied a multiple linear regression to assess the influence of variables such as age, fatigue, and depression on the correlation between the SDMT and the PASAT3 and the other cognitive tests.

A receiver operating characteristic (ROC) curve for SDMT and PASAT3 was also generated to obtain sensitivity and specificity values. The same procedure was followed to identify which test was more sensitive to detect changes in cognitive impairment one year later.

Statistical analysis was performed using SPSS 22.0 (IBM, New York, NY) and a p value <0.05 was considered statistically significant.

## Results

### Demographic features

The group included 237 RRMS patients and 57 healthy controls. Table 1 shows the demographic and clinical data for both groups.

**Table 1:** **Demographic and clinical data of healthy controls and patients**

	**Healthy controls**	**Patients**	**p value**
Gender (male/female)	13/44	80/157	0.090
Age (mean ± SD; years)	40.5 ± 9.4	38.5 ± 10.2	0.198
Education (mean ± SD; years)	14.1 ± 3.4	13.1 ± 4.1	0.095
Disease duration (mean ± SD; years)	_	7.4 ± 7.1	
EDSS (median, range)	_	1.5 (0 – 6)	

### Cognitive performance: control group and patients

The control group had significantly higher scores in almost all cognitive variables assessed in the study (p <0.05), except for the STROOP interference score and the perseverative and non-perseverative errors on the WCST. Scores of self-reported inventories of fatigue and depression were significantly higher in the patient group (p <0.001). Results are summarized in Table 2.

**Table 2:** **Neuropsychological assessment data for healthy controls and patients**

	**Healthy controls**	**Patients**	**p value**
	**Mean (SD)**	**Mean (SD)**	
SRT-Storage	50.9 (8.8)	45.9 (13.6)	0.001*
SRT-Retrieval	43.2 (11.1)	37.1 (13.9)	0.002*
SRT-Delayed	9.7 (1.7)	8.7 (2.4)	0.003*
10/36	22.5 (4.5)	20.6 (5.2)	0.015*
10/36 delayed	8.0 (1.9)	7.3 (2.2)	0.017*
SDMT	63.7 (8.6)	54.3 (13.4)	<0.001*
PASAT 3 seconds	48.6 (8.3)	44.8 (11.4)	0.005*
PASAT 2 seconds	39.3 (7.3)	36.2 (10.3)	0.086
Semantic verbal fluency	21.5 (3.8)	19.1 (4.4)	<0.001*
Phonetic verbal fluency	17.2 (3.6)	14.7 (4.3)	<0.001*
STROOP word	44.7 (9.5)	40.6 (8.7)	0.003*
STROOP color	46.9 (8.0)	42.0 (8.3)	0.001*
STROOP word-color	49.8 (9.2)	44.9 (9.2)	0.001*
STROOP interference	53.3 (9.0)	52.1 (8.2)	0.323
WCST perseverative errors	12.2 (17.2)	14.0 (12.5)	0.383
WCST no perseverative errors	18.1 (8.4)	18.8 (9.6)	0.585
WCST categories	5.3 (1.1)	4.8 (1.7)	0.025*
Line orientation test	26.5 (2.6)	25.1 (3.3)	0.004*
WAIS matrix subtest	11.6 (2.5)	10.3 (2.4)	<0.001*
RBMT	10.2 (1.3)	9.6 (1.8)	0.021*
FSS	2.2 (0.9)	3.7 (2.0)	<0.001*
BDI	3.7 (3.7)	9.1 (7.6)	<0.001*

*p < 0.05 was considered statistically significant.

### SDMT and PASAT3 comparison

Thirty-one (13.1%) patients and 3 (5.3%) controls were not willing to perform PASAT3 whereas all participants completed the SDMT. Patients who did not complete the test were older and had fewer years of education. These patients had a longer duration of disease, were treated with disease modifying therapies for longer periods, and had higher EDSS scores. No statistically significant differences were observed regarding time since the last relapse. The Beck Depression Inventory score was higher in the group that did not complete PASAT3. No significant differences were observed in the Fatigue Severity Scale score. Table 3 shows the demographic features of patients who completed the PASAT3 and those who did not.

**Table 3:** **Demographic characteristics of patients who completed the PASAT3 and those who did not**

	**PASAT3**	**PASAT3**	**p value**
	**Completed**	**Not completed**	
	**Mean (SD)**	**Mean (SD)**	
Age	38.2 (9.9)	44.4 (9.5)	0.001*
Educational level	13.5 (3.8)	11.3 (4.9)	0.003*
Years of disease evolution	6.7 (6.6)	12.1 (8.3)	<0.001*
Total time of treatment	1.6 (2.7)	3.1 (3.2)	0.019*
Time since last relapse	1.4 (2.3)	2.7 (3.6)	0.064
FSS	3.3 (1.8)	3.8 (2.2)	0.297
BDI	7.5 (6.9)	11.4 (8.6)	0.005*
EDSS	1.8 (1.5)	2.4 (1.4)	0.028*

*p < 0.05 was considered statistically significant.

The Pearson correlation coefficient showed a positive correlation between PASAT3 and SDMT (r = 0.608, p <0.001). Both tests showed a significant correlation with other neuropsychological tests. Nevertheless, although differences were not statistically significant, correlation values were higher for SDMT for almost all cognitive tests and for variables such as fatigue, depression, and EDSS.

When controlling for age, fatigue, and depression, multiple linear regression showed that coefficients between SDMT and PASAT3 and other cognitive tests were generally lower, but again, most were higher for SDMT. All tests remained significant. Data are shown in Table 4.

**Table 4:** **Correlation coefficients and significance of SDMT and PASAT3 when controlling for age, fatigue, depression**

	**SDMT (** ***r*** **)**	**SDMT (** ***p*** **)**	**PASAT3 (** ***r*** **)**	**PASAT3 (** ***p*** **)**
SRT-Storage	0.409	<0.001*	0.306	<0.001*
SRT-Retrieval	0.472	<0.001*	0.370	<0.001*
SRT-Delayed	0.451	<0.001*	0.219	<0.001*
10/36	0.377	<0.001*	0.430	<0.001*
10/36 delayed	0.332	<0.001*	0.380	<0.001*
Semantic verbal fluency	0.375	<0.001*	0.303	<0.001*
Phonetic verbal fluency	0.401	<0.001*	0.436	<0.001*
STROOP word	0.386	<0.001*	0.304	<0.001*
STROOP color	0.552	<0.001*	0.537	<0.001*
STROOP word-color	0.512	<0.001*	0.465	<0.001*
STROOP interference	0.228	<0.001*	0.231	0.001*
WCST perseverative errors	−0.229	0.001*	−0.286	<0.001*
WCST no perseverative errors	−0.208	0.002*	−0.281	<0.001*
WCST categories	0.220	0.001*	0.261	0.001*
Line orientation Test	0.331	<0.001*	0.238	0.001*
WAIS matrix subtest	0.417	<0.001*	0.435	<0.001*
RIVERMEAD	0.435	<0.001*	0.419	<0.001*

*p < 0.05 was considered statistically significant.

### Sensitivity and specificity of SDMT and PASAT3

**Figure 1:**
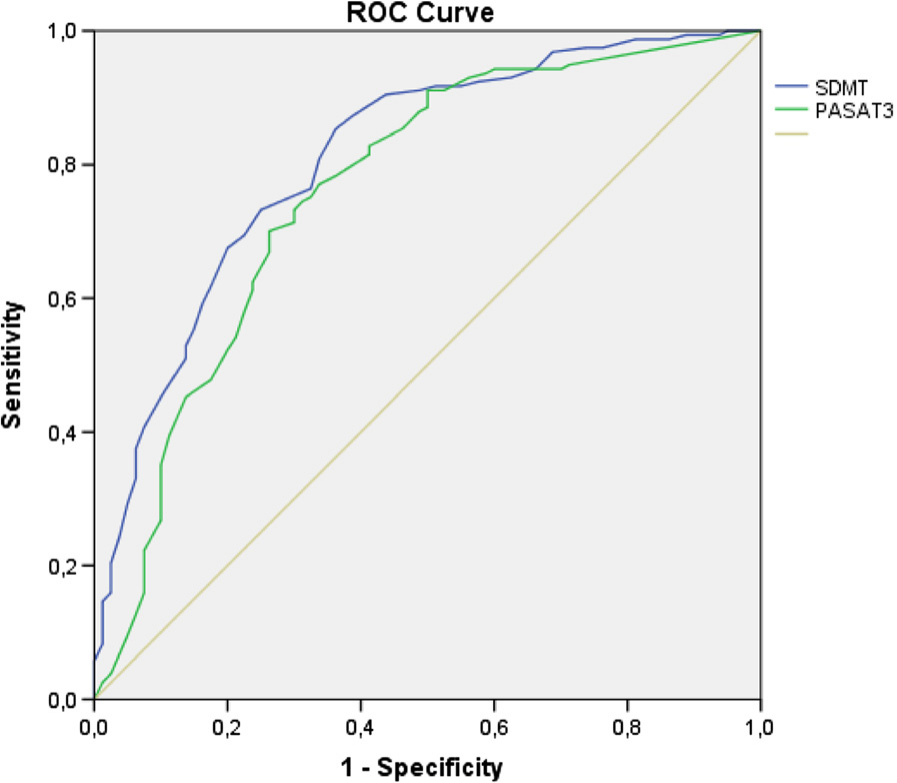
**ROC curve for SDMT and PASAT3 (first assessment).**

In this sample, 33.8% of patients were classified as CI. According to the definition of CI, SDMT had a sensitivity of 0.809 and a specificity of 0.662, while PASAT3 had a sensitivity of 0.783 and a specificity of 0.637. The area under the curve was 0.811 for SDMT (confidence interval: 0.752–0.870) and 0.761 for PASAT3 (confidence interval: 0.693–0.830). A cut-off score of 49 for SDMT classified CI patients with an accuracy of 75%, compared with a cut-off score of 35 and an accuracy of 73% for PASAT3. Figure 1 shows the ROC curve.

**Figure 2:**
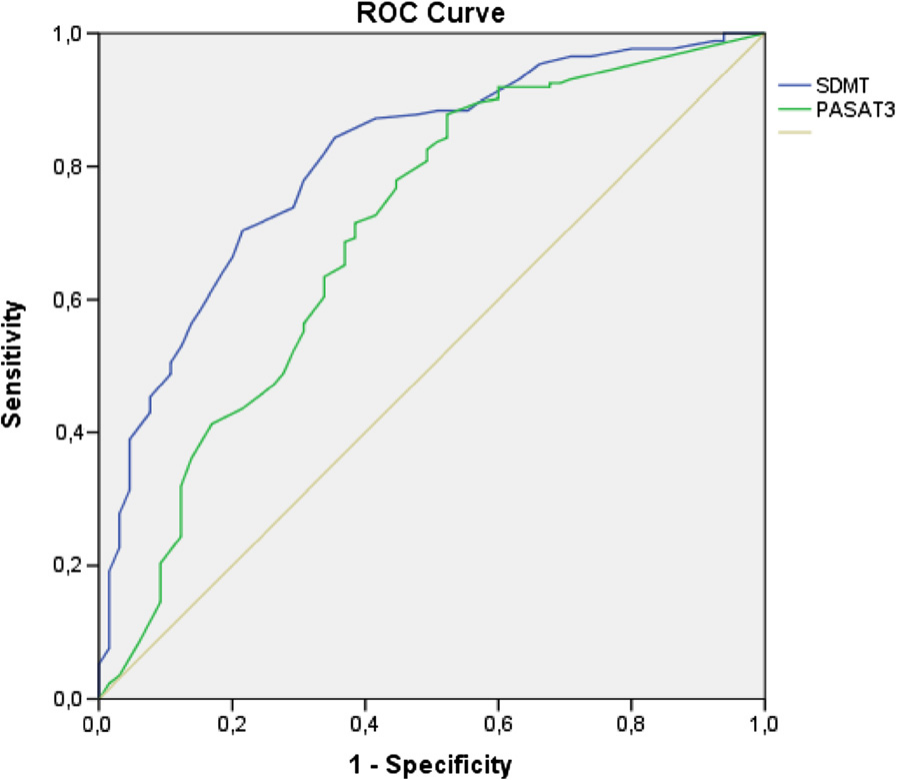
**ROC curve for SDMT and PASAT3 (first assessment, excluding SDMT and PASAT).**

In the previous analysis, both SDMT and PASAT3 were part of the gold standard for detecting CI in MS patients. Therefore, we performed another analysis using the same criteria of failure in at least two tests but excluding SDMT and PASAT3. We found that 27.4% of patients were classified as CI. SDMT had a sensitivity of 0.779 and a specificity of 0.692, while PASAT3 had a sensitivity of 0.727 and a specificity of 0.585. The area under the curve was 0.809 for SDMT (confidence interval: 0.749–0.870) and 0.700 for PASAT3 (confidence interval: 0.620–0.779). A cut-off score of 49 for SDMT classified CI patients with an accuracy of 75%. A cut-off score of 35 for PASAT3 classified CI patients with an accuracy of 68%. Figure 2 shows the ROC curve.

**Figure 3:**
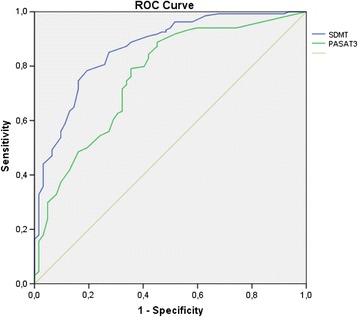
**ROC curve for SDMT and PASAT3 (second assessment).**

One hundred and ninety-six patients were assessed one year later. The proportion of CI patients in the baseline assessment in this group was 27.6%. At one-year follow-up, the proportion of CI subjects increased to 31.6% with the BRNB battery. SDMT had a sensitivity of 0.824 and a specificity of 0.741, while PASAT3 had a sensitivity of 0.796 and a specificity of 0.684. The area under the curve was 0.860 for SDMT (confidence interval: 0.802–0.918) and 0.772 for PASAT3 (confidence interval: 0.690–0.853). A cut-off score of 49 for SDMT classified CI patients with an accuracy of 80% compared with a cut-off score of 35 for PASAT3, which had an accuracy of 73%. Figure 3 shows the ROC curve.

**Figure 4:**
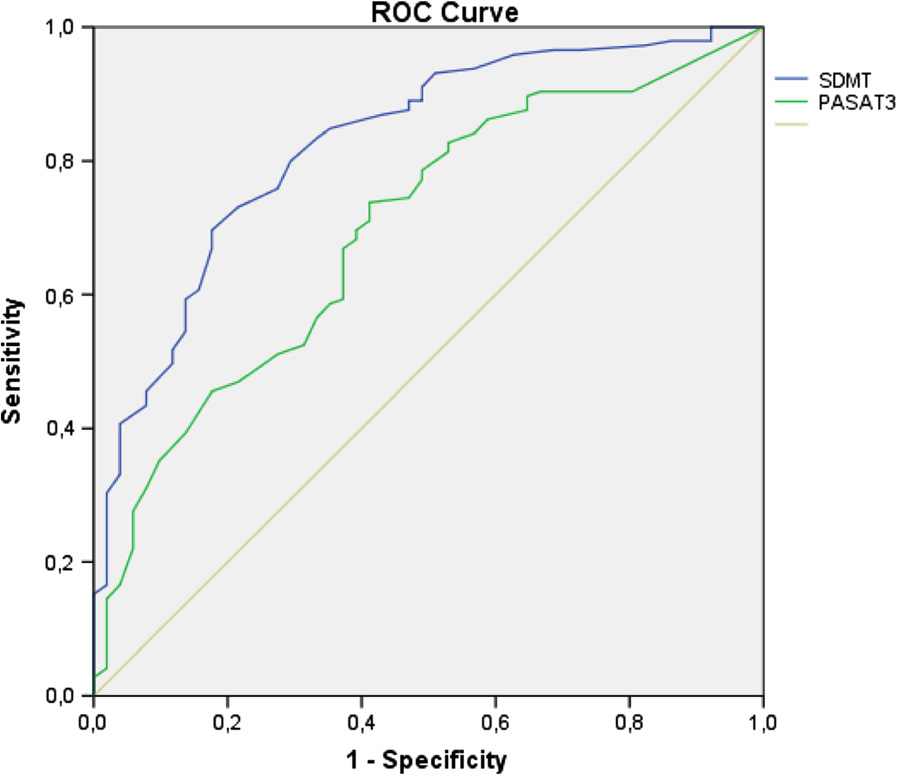
**ROC curve for SDMT and PASAT3 (second assessment, excluding SDMT and PASAT).**

The same analysis was performed without SDMT and PASAT3 as part of the gold standard. According to these criteria, 25% of patients in the baseline assessment in this group were CI. At one-year follow-up, this percentage increased to 26%. SDMT had a sensitivity of 0.800 and a specificity of 0.706, while PASAT3 had a sensitivity of 0.738 and a specificity of 0.588. The area under the curve was 0.826 for SDMT (confidence interval: 0.762–0.891) and 0.697 for PASAT3 (confidence interval: 0.616–0.779). A cut-off score of 49 for SDMT classified CI patients with an accuracy of 77%, and a cut-off score of 35 for PASAT3 classified CI patients with an accuracy of 68%. Figure 4 shows the ROC curve.

Fatigue and depression are common symptoms in MS that can affect cognitive function; therefore, we assessed the sensitivity and specificity of SDMT and PASAT taking into account these two factors. We found 50% of patients had symptoms of fatigue and 32.5% had symptoms of depression. When we compared the fatigue group (FSS >3) with the non-fatigue group, and the depressed group (BDI >11) with the non-depressed groups, the area under the curve was higher for patients without fatigue or depressive symptoms.

A t-test for repeated measures between the first and the second evaluation showed that the mean score of SDMT was 54.5 at the first assessment and 55 at the second assessment. This difference was not significant (p = 0.308). The mean score of PASAT3 was 45.2 at the first assessment and 46.7 at the second. This was statistically significant (p = 0.011), suggesting a higher learning effect in PASAT3 than in SDMT.

## Discussion

This study showed that both SDMT and PASAT3 could detect CI in a significant portion of MS patients but suggests that SDMT has a better ability to detect CI after a year of follow-up. We found significant correlations between PASAT3 and SDMT with measurements of memory and executive function. Correlation values were uniformly higher for SDMT, but were not statistically significant. Parmenter et al. [10] also reported significant correlations between SDMT and neuropsychological performance. These data support previous findings regarding the use of these two tests as cognitive assessments to detect CI in MS.

Previous studies suggested the importance of comparing SDMT and PASAT and defining which of the two is more specific and sensitive as a measure for CI [10]. According to our definition of CI, sensitivity values for both SDMT and PASAT3 were similar, although those for SDMT were slightly higher. A cut-off score of 49 for SDMT and of 35 for PASAT3 classified CI patients with an accuracy of 75 and 73%, respectively. Thus, both tests may be used as screening or monitoring tools because they measure the speed of information processing and working memory. The proportion of CI patients in our sample was similar to that in previous reports [28,29].The area under the curve was slightly greater for SDMT than for PASAT3 at the baseline evaluation and after one year of follow-up. Similar results were obtained when we analyzed data without SDMT and PASAT3 as part of the gold standard and when factors such as fatigue and depression were considered. Although they did not reach statistical significance, this might indicate that SDMT performs better than PASAT3 as a test to assess CI.

Our finding that all patients completed SDMT but that 31 (13.1%) patients were not willing to perform PASAT3 is in agreement with a previous study that reported missing values for PASAT3 [15] and another that reported that 17% of the subjects refused to attempt PASAT [6]. Performance in PASAT3 was influenced by patient age, duration of the disease, and degree of disability measured by EDSS. Besides the patients’ basal characteristics, anxiety may also affect performance in PASAT. Glanz et al. [30] described an association between PASAT and patient anxiety and Tombaugh [31] also reported that PASAT created anxiety and frustration. In the control group, 3 subjects (5.3%) decided not to complete PASAT3 even though they completed SDMT. These data support previous findings in a general population regarding difficulties in answering items in PASAT, even in ideal conditions [14].

When we compared factors related to the administration of SDMT and PASAT3, we found that SDMT was completed in a shorter time and that the examples and the instructions were more easily understood and followed by patients during the task. Furthermore, previous studies reported that only minimal training was needed to master the administration of SDMT [32] and that it could be completed in a very short time (5 minutes) [10]. Importantly, no electronic devices are needed to administer the test.

When administering PASAT3, it was sometimes necessary to repeat the instructions more than once, making the test longer. Some patients decided not to continue the test as they complained of difficulties adding the numbers. An additional inconvenience of PASAT is that performance is affected by mathematical ability [31,33].

Previous studies showed that SDMT is sensitive for the detection of cognitive deterioration over time, has a moderated practice effect [26] and is a reliable test over multiple test-retest intervals [32]. PASAT showed a transient practice effect in a 5-year follow-up study, with relatively stable results after the second evaluation [34]. In our sample, differences in the mean score between the first and second evaluation were not statistically significant by SDMT while they were significant by PASAT3. These data are in accordance with previous findings in which patients set up strategies to answer the test, ignoring some items that interfered with the functions it aimed to measure [33].

A limitation of our study is that the low conversion rate to CI in MS over a year might have influenced the ability of SDMT and PASAT3 to detect incident cases of CI. However, our study has several strengths. Both depression and fatigue were taken into account, factors that have not been assessed in previous studies but which may influence cognitive performance [15]. Furthermore, we selected RRMS patients so as to assess the utility of SDMT and PASAT in a less impaired and more homogeneous group that better represents the initial phases of MS. Finally, we performed a follow-up visit in our patients one year after the first evaluation. In previous studies, follow-up visits were carried out at different times.

## Conclusions

We found that even though the differences in sensitivity between SDMT and PASAT3 were minor, the differences were uniformly in the direction of SDMT having slightly better validity, both at baseline and 1-year follow-up. In addition, SDMT was easier and faster to administer compared with PASAT3, and had a greater correlation with other cognitive tests in the presence of fatigue or depression.
